# Alphaviruses in Gene Therapy

**DOI:** 10.3390/v1010013

**Published:** 2009-04-21

**Authors:** Kenneth Lundstrom

**Affiliations:** PanTherapeutics, Rue des Remparts 4, CH1095 Lutry, Switzerland; E-Mail: lundstromkenneth@googlemail.com; Tel.: +41 79 776 6351

**Keywords:** Viral vectors, Vaccines, CNS, Cancer therapy

## Abstract

Alphaviruses are enveloped single stranded RNA viruses, which as gene therapy vectors provide high-level transient gene expression. Semliki Forest virus (SFV), Sindbis virus (SIN) and Venezuelan Equine Encephalitis (VEE) virus have been engineered as efficient replication-deficient and -competent expression vectors. Alphavirus vectors have frequently been used as vehicles for tumor vaccine generation. Moreover, SFV and SIN vectors have been applied for intratumoral injections in animals implanted with tumor xenografts. SIN vectors have demonstrated natural tumor targeting, which might permit systemic vector administration. Another approach for systemic delivery of SFV has been to encapsulate replication-deficient viral particles in liposomes, which can provide passive targeting to tumors and allow repeated administration without host immune responses. This approach has demonstrated safe delivery of encapsulated SFV particles to melanoma and kidney carcinoma patients in a phase I trial. Finally, the prominent neurotropism of alphaviruses make them attractive for the treatment of CNS-related diseases.

## 1. Introduction

Gene therapy has demonstrated some genuine promise during the last decade. For instance, the cure of severe combined immune deficiency (SCID-X1) patients with retrovirus vectors [1] can be considered a real success, despite the severe adverse events occurring in some patients [2]. These unexpected setbacks relate to the novelty of gene therapy and the shortcomings need to be addressed through intensive research. Other applications have been in the area of cancer, hemophilia and CNS disorders [3]. Adenoviruses are commonly used viral vectors [4]. Moreover, a number of clinical studies have also been conducted with adeno-associated virus (AAV) [5]. Furthermore, retroviruses, herpes simplex virus, vaccinia virus and more recently lentiviruses have been commonly utilized in animal models [6]. However, alphaviruses have not been explored in large extent for gene therapy applications, so far. Their special features and potential are described below.

**Figure 1.**
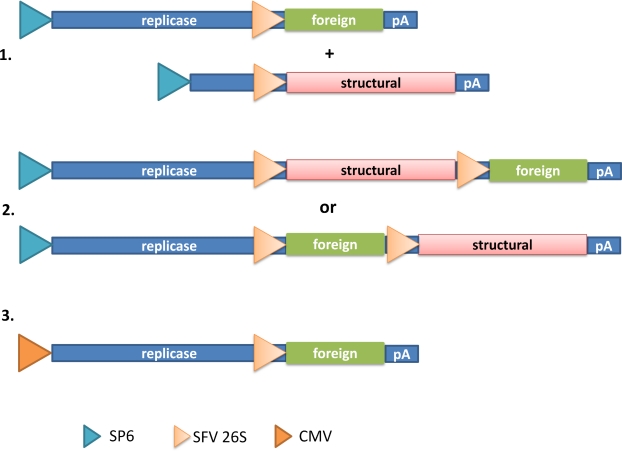
Alphavirus expression systems. (1.) Replication-deficient vector system: Two vectors (expression and helper vectors) are required for the generation of recombinant particles. The SP6 RNA polymerase promoter is utilized for *in vitro* RNA transcription and the subgenomic 26S promoter for transgene and structural gene expression. (2.) Replication-competent vector system: A single vector with the full-length genome and an additional 26S subgenomic promoter drives the expression of the foreign gene. The foreign gene can be placed upstream or downstream of the structural genes. (3.) DNA-RNA layered vector system: A single vector where the SP6 RNA polymerase promoter has been replaced by a CMV promoter allows a direct transient transfection approach.

Alphaviruses are single-stranded RNA (ss-RNA) viruses with positive polarity [7]. They possess an envelope consisting of two or three major proteins forming heterologous spikes. Among the many alphavirus members, Semliki Forest virus (SFV) [8], Sindbis virus (SIN) [9] and Venezuelan Equine Encephalitis (VEE) virus [10] have been engineered as efficient delivery and expression vectors. The following types of vectors have been constructed (Figure 1): 1. Replication-deficient vector system, which usually comprises of two vectors, expression and helper vectors, although using split helper vectors require the use of two helper vectors [11]. The expression vector contains the viral nonstructural genes responsible for replicase function of RNA. The structural genes have been deleted and replaced by the foreign gene of interest. Co-transfection with a helper vector containing the structural genes will provide the means for production of virus particles. However, due to the presence of the RNA packaging signal only on the expression vector, the generated virus particles will be replication-deficient. Although no new virus progeny will be generated upon infection with these particles, they will generate high level transgene expression in target cells. 2. Replication-proficient vector system, which contains the full-length alphavirus genome and the additional foreign gene of interest. These vectors provide heterologous gene expression as well as generation of new virus progeny. The advantage of replication-proficient vectors is their prolonged expression profile and possibility to extend the infection to neighboring cells *in vivo*. On the other hand, these vectors pose a greater safety risk. 3. Layered DNA-RNA vector system has the SP6 RNA polymerase promoter replaced by a CMV promoter, which will allow direct application of the vector as a transfection agent. Due to the presence of the SFV replicase genes, the extensive RNA replication will result in superior gene expression compared to conventional plasmid vectors. Moreover, as no virus particles are produced at any stage, the safety aspects are very high. In contrast, relying on only transfection methods significantly compromises the gene delivery efficacy.

Alphavirus vectors have been used in a number of applications [12]. Recombinant protein expression has been established for activity and functionality determinations and a special emphasis has been dedicated to membrane proteins. Specifically, SFV vectors have been employed for the expression of G protein-coupled receptors (GPCRs) and ion channels in various mammalian cell lines [13]. Moreover, the high infection rate of neuronal cells has made alphavirus vectors attractive tools in neuroscience. In this context, studies on location, transport and function of recombinant proteins have been conducted in primary neurons [14] as well as in hippocampal slice cultures [15]. Moreover, intracranial administration resulted in local transient reporter gene expression with no visible side effects in rodents [16]. Large-scale protein production in mammalian suspension cultures in spinner flasks and bioreactors has provided recombinant GPCRs for drug screening projects and structural biology. For instance, in a structural genomics initiative, 101 GPCRs were expressed in 3 mammalian cell lines from SFV vectors [17]. In this review, the focus will be on the application of alphavirus vectors for gene therapy.

## 2. Alphaviruses in Gene Therapy

Alphaviruses have been used for three main applications in gene therapy. Their transient expression nature has made them more suitable for applications where only short-term expression is required. In this context, high expression with the subsequent disappearance of the vector is a preferred feature. Typically, alphavirus vectors have been employed for vaccine production. In this application it is certainly an advantage to provide high levels of transient gene expression. Likewise, the demands for successful cancer therapy are short-term but high level expression of the therapeutic gene of interest as continuous presence of actively expressed therapeutic is undesirable. The third area of interest is the treatment of central nervous system (CNS) diseases, as alphaviruses have a strong preference for neuronal cells. However, as many CNS-related diseases require long-term or even life-long therapeutic effect the transient expression mode of alphaviruses is not ideal. The recent engineering of replication-competent oncolytic viruses may address this question to some extent.

### 2.1. Vaccines

Application of alphavirus vectors for vaccine generation has included SFV, Sindbis virus and VEE virus administered as replication-deficient virus particles, naked RNA and layered DNA-RNA vectors [18]. The main application has been for vaccine development against viruses in which vaccination with various viral surface expressing antigens has resulted in immunity and protection against challenges against lethal doses of pathogenic viruses in animal models. Additionally, a number of studies have described tumor vaccine development [19–24] (Table I). In this context, all three types of expression vectors have been applied. Different target genes such as interleukin-12 (IL-12) [19], human papilloma virus (HPV) E6 and E7 [20, 21], P815A tumor antigen [22] and the PIA gene [25] have been used. Interestingly, vaccination with naked SFV RNA replicons expressing bacterial β-galactosidase led to therapeutic efficacy and even protection in vaccinated mice [26]. As little as 1 μg of SFV-LacZ RNA provided complete tumor protection and even when tumors were injected two days prior to immunization, the survival of mice was extended by 10–20 days compared to control animals [26]. Likewise, protection against P185 tumor challenges was obtained after vaccination with SFV particles expressing the P1A gene [25]. Mice were also protected from new tumor development after immunization with VEE vectors expressing the HPV-E7 gene [27]. An interesting approach was to subject isolated dendritic cells from mouse bone marrow to SFV particles expressing B16 and 203 tumor antigens, which demonstrated protection against B16 and 203 gliomas and also prolonged the survival of animals with established tumors [28]. The DNA-RNA layered Sindbis vector was used to overexpress the murine melanoma cell adhesion molecule (MCAM/MUC18) for vaccination against B16F10 murine melanoma cells [29]. The vaccination led to efficient protection of mice from lethal challenges with melanoma-expressing murine MUC18 in both primary and metastatic tumor models. This could present an excellent strategy for the future prevention and treatment of melanoma. In another application, VEE particles carrying the truncated neu oncogene were used to transduce dendritic cells (DCs), which induced robust neu-specific CD8^+^ T-cell and anti-neu IgG responses [30]. It was also demonstrated that a single administration of VEE-transduced DCs resulted in the regression of large established tumors in mice. VEE particles have also been applied for prostate-specific membrane antigen (PSMA) immunotherapy [31]. Mice vaccinated with VEE-PSMA particles showed robust T-cell and B-cell responses after a single injection and anti-PMSA were detected after three immunizations. VEE-PSMA particles induced more immunogenic responses than purified PSMA protein-adjuvants. Recently, it was demonstrated in transgenic mice, tolerant for HPV16-E6E7, that SFV-IL12 also stimulated SFV-HPV E6-E7-induced CTL responses [32].

### 2.2. Cancer Therapy

The main gene therapy applications of alphaviruses have dealt with cancer animal models. In this context, the most popular approach has been intratumoral injection of alphavirus vectors carrying reporter and/or therapeutic genes. For instance, SFV vectors expressing the p40 and p35 subunits of interleukin-12 (IL-12) showed significant tumor regression and inhibition of tumor blood vessel formation in a murine B16 tumor model [33]. Furthermore, SFV-GFP particles demonstrated substantial reduction in immunodeficient mice in implanted human lung tumors after intratumoral injections [34] indicating that the SFV particles as such had therapeutic effect. This finding was confirmed by the treatment of rapidly growing K-BALB and CT26 tumors with SFV particles, which resulted in apoptosis induction [35]. The expression of IL-12 [36] and IL-18 [37] from SFV vectors resulted in complete regression of K-BALB tumors in BALB/c mice and therapeutic efficacy in CT26 tumors, respectively. In these studies, an SFV vector containing the translation enhancement signal from the capsid gene was applied to provide 10 times higher transgene expression. In another application, the expression of the murine vascular endothelial growth factor receptor-2 (VEGF-2) from SFV vectors led to the inhibition of angiogenesis, which reduced tumor growth and metastasis in mice [38].

### 2.3. Oncolytic Vectors

Replication-competent alphaviruses have been engineered in attempts to prolong the effect of transgene expression and to enhance distribution *in vivo*. The avirulent SFV strain A7(74) has been the basis for the VA7-EGFP construct as illustrated in Figure 1. It was demonstrated that VA7-EGFP efficiently infected and lysed a number of cancer cell lines [39]. In SCID mice grafted with human melanomas a single injection (intratumoral, intraperitoneal or intravenous) of 10^6^ pfu virus resulted in significant tumor regression. Not surprisingly, histological examination showed that in addition to tumor tissue the virus was present in normal tissue including the brain. The oncolytic VA7-EGFP vector was evaluated in human osteosarcoma xenografts in a nude mouse model [40]. Treatment with VA7-EGFP resulted in significant regression of tumors. In an orthotopic osteosarcoma nude mouse model with highly aggressive tumor growth oncolytic SFV extended the survival although no animal was finally cured. The oncolytic VA7-EGFP virus was also evaluated in an orthotypic lung cancer tumor model in nude mice [41]. In this study, VA7-EGFP was capable of providing a significantly increased survival rate comparable to achievements with a conditionally replicating Adenovirus (Ad5-Δ 24TK-GFP). However, the systemic administration of oncolytic virus demonstrated limited efficacy and requires further improved vectors.

### 2.4. CNS Applications

The neurotropic nature of alphaviruses has made them attractive vectors for the treatment of CNS-related diseases. However, the neurovirulence of many SFV vectors has limited their applications in the CNS and has switched the focus to avirulent strains [42]. In this context, it was shown that VA7-EGFP (based on the avirulent strain A7(74) resulted in reduced GFP expression in neurons, but enhanced presence in glial cells [43]. *In vivo* studies in rat [14] and mouse [44] brain demonstrated transient local reporter gene expression after stereotactic injections into amygdala, striatum and substantia nigra. However, in the mouse brain cell degeneration and axonopathy [44], an indication of neuronal loss, was observed only for the wild type SFV vector, suggesting that less cytotoxic vectors such as A7(74) are required for successful CNS gene therapy.

SFV vectors have been evaluated in experimental autoimmune encephalomyelitis (EAE) in the mouse CNS induced by injection of myelin as an animal model for multiple sclerosis. Previously, it had been shown that SFV is capable of infecting the CNS via the olfactory bulb after intranasal delivery [45]. Balb/c mice subjected to intranasal administration of SFV-EGFP particles demonstrated reporter gene expression in the olfactory bulb already after one day and it lasted for seven days [46]. When mice induced for EAE were intranasally injected with SFV particles expressing IL-10, the clinical EAE was significantly reduced whereas SFV-EGFP and empty SFV vectors exacerbated the disease. In another study, SFV particles were employed for intranasal administration of interferon β (IFN β) [47]. In this case, inhibition of EAE was dependent on the timing of SFV administration and the number of injections. Furthermore, the avirulent SFV strain A7(74) was applied for the expression of the transforming growth factor β [48] and the inhibitor of metalloproteinase 2 [49] in mice. Despite intraperitoneal administration, the vector was able to cross the blood-brain barrier and demonstrated efficacy in inhibiting EAE.

### 2.5. Mutant Vectors

Several attempts have been made to further improve alphavirus vectors especially addressing the cytotoxicity of existing vectors and relatively short duration of transgene expression. In this context, Sindbis virus vectors with point mutations in the nonstructural gene nsP2 were shown to significantly reduce the cytotoxic effect on host cells [50, 51]. The point mutation P726S in nsP2 not only reduced the cytoxicity, but also enhanced expression levels. Furthermore, the combination of the P726S mutation with a point mutation in nsP4 resulted in a less cytotoxic and temperature-sensitive phenotype [52]. Similarly, point mutations introduced into the SFV vector has managed to reduce the cytoxicity. For instance, two point mutations in nsP2 (S259P, R650D) resulted in substantially reduced cytotoxicity and enhanced transgene expression [53]. A point mutation at position 713 in nsP2 produced a replication-persistent phenotype in BHK cells [54]. The combination of the S259P, R650D and L713P resulted in reduced cytotoxicity, temperature-sensitivity and prolongation of gene expression [55]. Also, the combination of the mutations R649H and P718T generated an SFV vector which showed 100-fold lower RNA levels compared to wildtype vector although the β-gal levels were similar [56].

As described above, the nonstructural genes (nsP1-4) of the avirulent SFV strain A7(74) have been the basis of an expression vector, which showed a temperature-sensitive phenotype in BHK cells [43]. Moreover, studies in hippocampal slice cultures demonstrated highly specific expression in glial cells at 37°C, but reporter gene expression almost uniquely in neurons at 31°C.

### 2.6. Targeting of Viral Particles

The broad host range of alphaviruses and not the least due to their preference to target the CNS [6] has resulted in attempts to target the virus to the therapeutic cell/tissue of interest. Already more than 10 years ago chimeric Sindbis virus particles were engineered by the introduction of IgG binding domains of protein A in the E2 Sindbis envelope [57]. This approach resulted in a 10^5^-fold reduction of infection efficacy of BHK cells, but allowed enhanced infection rates of cells treated with a monoclonal antibody against surface proteins. More recently, it was discovered that Sindbis virus possesses natural targeting to tumors [58]. Intraperitoneal injections showed specific reporter gene expression in implanted tumors in mice. Moreover, subcutaneous daily injections of SIN-IL12 virus reduced the tumor load to 6.2% of control animals. Most astonishingly, intraperitoneal injections of SIN-Luc virus resulted in tumor targeted expression in spontaneous fibrosarcomas in the mouse tail.

As no similar natural targeting to tumors has been observed for SFV, another approach to achieve targeting has been to encapsulate SFV particles in liposomes [59]. Efficient encapsulation of SFV-LacZ particles was obtained and systemic delivery in SCID mice with implanted tumors showed targeted β-gal expression in tumors. The systemic delivery was demonstrated safe by extensive histological and toxicological analysis. These findings set the basis for a clinical trial applying encapsulated SFV-particles as described below.

### 2.7. Clinical Trials for Alphaviruses

Alphaviruses have obviously been used to a lesser extent in clinical trials compared to for instance retrovirus and adenovirus vectors. Despite this, a number of clinical trials have been completed or initiated [60]. Most of these trials are phase I safety trials in healthy volunteers using VEE replicons. AlphaVax has sponsored one vaccine trial for Cytomegalovirus (CMV) expressing the CMV proteins gB, pp65 and IE1. In another study, the influenza hemagglutinin (HA) protein was expressed from VEE vectors and evaluated as an influenza vaccine. The National Institute for Allergies and Infectious Diseases (NIAID) has sponsored a safety study on an HIV gag vaccine expressed from the VEE vector in healthy uninfected volunteers. Currently, recruiting is in progress for an immunotherapy study with VEE-CEA(6D) vectors in patients with advanced or metastatic CEA expressing malignancies.

A phase I clinical trial in melanoma and kidney carcinoma patients was conducted with encapsulated SFV particles expressing the p40 and p35 subunits of IL-12 [61]. The study showed no liposome- or SFV-related toxicity. The maximum tolerated dose (MTD) was determined to 3 × 10^9^ encapsulated particles per m^2^, which was strongly influenced by the high transient expression of recombinant IL-12 resulting in a fever response. The plasma IL-12 levels showed a 5-fold increase, which lasted for 4–7 days. Due to the liposome-encapsulation, SFV particles were not recognized by the host immune defense system and allowed therefore repeated systemic administration. The phase I study clearly demonstrated that encapsulated SFV vectors can be safely administered systemically to cancer patients. Despite the protocol for a phase I/II trial for the treatment of recurrent glioblastoma multiforme with encapsulated SFV vectors published in 2003 [62], the study has not been conducted.

## 3. Conclusions

Alphavirus vectors have showed versatility in gene therapy applications. The most frequently used vectors are based on SFV, Sinbis and VEE. In the future, also other alphaviruses might be developed into functional expression vectors. The replication-deficient, replication-competent and DNA-RNA layered vectors provide alternative delivery approaches. Furthermore, naked RNA has been shown feasible for immunization in vaccine development. Alphaviruses have been used for vaccine development approaches to establish both therapeutic and prophylactic efficacy against tumors. Moreover, alphavirus vectors have been evaluated in a variety of animal tumor models by intratumoral, intraperitoneal and intravenous administration. Efforts have been taken to target vectors to tumors by introduction of targeting sequence in the viral envelope or by encapsulation of viral particles in liposomes. Due to the efficient CNS delivery, alphavirus vectors have been evaluated to a limited extent as therapeutic vectors in animal models of CNS disease. There are certainly some limitations related to the applications of alphavirus vectors. Their transient expression profile makes it difficult to foresee any serious treatment of chronic diseases, where long-term or even life-long gene expression is generally required. The cytotoxic effect of alphaviruses must also be considered as a limitation, although it can be turned into an advantage in cancer therapy. The broad host range and especially the strong neurotropism certainly raise questions of concern about safety. Moreover, the lack of appropriate packaging cell lines will limit GMP production for clinical trials.

For future applications of alphaviruses as gene therapy vectors most of the limitations mentioned above, except long-term expression, can be addressed through additional research and development. Despite the engineering of a number of mutant alphavirus vectors with improved features compared to wild type vectors, there is still a possibility for additional improvement such as reduced cytotoxicity and prolonged expression profiles. Additional engineering of targeted alphavirus vectors will be beneficial. The engineering of an appropriate packaging cell line is necessary to ensure GMP production of vectors for clinical trials.

## Figures and Tables

**Table 1.** Application of alphavirus vectors for generation of tumor vaccines.

**Target**	**Gene**	**Vector/Delivery**	**Response**	**Ref**
Brain tumor	IL-12	SFV/particles	Immunogenicity	[19]
Cervical cancer	HPV E6-E7	SFV/particles	Tumor protection	[20]
Glioma	B16, 203	SFV/particles	Tumor protection	[28]
Tumor	β-gal	SFV/RNA	Tumor protection	[26]
Tumor	HPV E7	VEE/particles	Tumor protection	[27]
Tumor	HPVE7-VP22	SIN/particles	CD8^+^ T-cell response	[21]
Tumor	P815A	SFV/particles	Tumor protection	[22]
Tumor antigen	MHC Class II	SFV/particles, DNA	Immunogenicity	[23]
Tumor antigen	P185	SFV/particles	CTL, tumor protection	[25]
Tumor antigen	Tyr-related prot-1	SIN/DNA	Antitumor activity	[24]
Melanoma	MUC18	SIN/DNA	Tumor protection	[29]
Tumor	Neu	VEE/particles	Tumor protection	[30]
Prostate cancer	PSMA	VEE/particles	Immunogenicity	[31]

β-gal, β-galactosidase; CTL, Cytotoxic T-lymphocyte activity; HPV, human papilloma virus; IL, interleukin; MHC, major histocompatibility complex; MCAM, melanoma cell adhesion molecule; PSMA, prostate-specific membrane antigen; SFV, Semliki Forest virus; SIN, Sindbis virus; VEE, Venezuelan equine encephalitis virus.
